# Aggregation-induced emission in luminescent metal nanoclusters

**DOI:** 10.1093/nsr/nwaa208

**Published:** 2020-08-28

**Authors:** Zhennan Wu, Qiaofeng Yao, Shuang-quan Zang, Jianping Xie

**Affiliations:** Department of Chemical and Biomolecular Engineering, National University of Singapore, Singapore; Department of Chemical and Biomolecular Engineering, National University of Singapore, Singapore; Green Catalysis Center, and College of Chemistry, Zhengzhou University, China; Department of Chemical and Biomolecular Engineering, National University of Singapore, Singapore; Joint School of National University of Singapore and Tianjin University, International Campus of Tianjin University, China

Few- to hundred-atom luminescent metal nanoclusters (NCs) protected by an organic monolayer have recently emerged as a novel class of chromophores, with facile preparation, ultrafine size, low toxicity, high renal clearance and excellent photostability [1]. Luminescent metal NCs hold great promise for broad applications in lighting, imaging, sensing and therapeutics [2]; however, the unsatisfactory emission intensity of metal NCs has constrained their further practical applications. In particular, the complexity, diversity and mutability in terms of their total structures preclude an in-depth understanding of their emission origin [1,3]. Therefore, there are very limited approaches and principles available for the desired improvements and tailoring of their luminescence performance.

In this context, inspired by the concept of aggregation-induced emission (AIE) first coined by Tang in 2001 to clarify the organic fluorophores involved in photophysical variation upon aggregation [4], we successfully designed a new family of ultrabright Au(0)@Au(I)-SR (SR: deprotonated thiol ligands) core-shell NCs in 2012, by preserving a high content of Au(I)-SR complexes in the protecting shell. Thereafter, AIE-type luminescent metal NCs were developed with markedly improved emission intensity, leading to an increasing number of studies in both the fundamental and practical sectors [5].

In sharp contrast to the structure of large-sized metal nanoparticles (>3 nm), in which the individual ligand is attached directly to the close-packed metal core substrate, the structure of metal NCs can be illustrated by a ‘divide-and-protect’ model (Fig. 1a–c) with the ‘staple motifs’ of metal(I)-ligand wrapping over the metal core [6,7]. The composition and structure of metal(I)-ligand motifs are diverse (e.g. from monomeric ML_2_ to heptameric M_7_L_8_ in the case of Au NCs, where M and L denote the metal and ligand, respectively) and mostly determined by curvature of the metal core. The AIE concept allows emission enhancement of metal NCs by effective restriction of intra-/intermolecular motion (i.e. vibration and rotation) of surface motifs, minimizing non-radiative decay [8]. Keeping this in mind, the AIE of metal NCs is, in essence, an affair closely related to the surface-emission state on the basis of ligand-to-metal charge transfer (LMCT) and/or ligand-to-metal-metal charge transfer (LMMCT), generating radiative relaxation via a metal-centered triplet [1,9]. Therefore, the AIE-type luminescent metal NCs feature long decay lifetime (μs-level), low emission energy (<2.2 eV) and large Stokes shifts (>100 nm), and their emission intensity is highly dependent on the features of their surface and interface [5,10].

**Figure 1.**
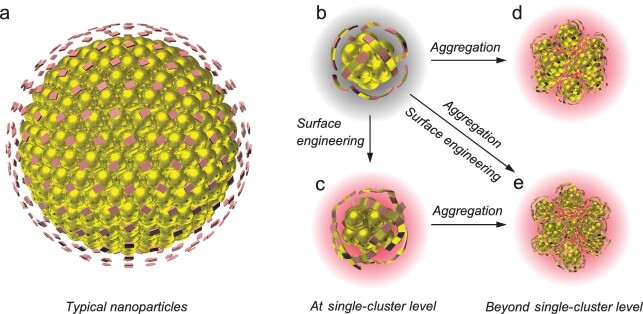
Schematic illustration of the typical structure model of organic layer-protected (a) metal nanoparticles (NPs) and (b and c) metal nanoclusters (NCs). In metal NPs, individual ligand is attached independently and directly to the close-packed metal core; whereas in metal NCs, unique staple-like metal(I)-ligand motifs (short and long/interlocked motifs for (b) and (c), respectively) wrap over the metal(0) core of metal NCs, following a ‘divide-and-protect’ model. (d) and (e) are the corresponding aggregates of (b) and (c), respectively. (Ligands: pink patches; metal(I): golden patches; metal(0): golden spheres; all hydrocarbon tails of ligands are omitted for clarity.)

This scenario imparts a fundamental principle for performance improvement of AIE-type luminescent metal NCs, that is to positively affect the LMCT and/or LMMCT process dictated by the landscape of the surface (a synergy from ligand-centered charge transfer process). In this vein and at a single-cluster level, several efficient strategies have been identified: 1) to strengthen metal(I)-ligand contacts by i) endowing surface ligands with increased electron donation capability to the core; ii) introducing electron-rich atoms or groups into surface ligands; and iii) increasing the content of metal(I) species in the surface motifs to strengthen the charge transfer process of LMCT and/or LMMCT; 2) to improve rigidity of the surface shell by i) constructing long and/or interlocked surface motifs (polymeric chain-like or ring-like metal(I)-ligand compounds) (Fig. 1c); and ii) conferring ligands with multiple interaction sites to anchor the metal core, and to enhance the rates of radiative energy transfer by suppressing ligand-related nonradiative relaxation of excited states. All these cases are related to controlling the excited state relaxation dynamics of LMCT and/or LMMCT with different emphases on each other, thus allowing production of brighter AIE-type luminescent metal NCs.

In addition, beyond the single-cluster level, treating metal NCs as building blocks to direct their aggregation is another well-recognized and effective strategy for rendering the strong luminescence of the metal NCs (Fig. 1d and e). Aggregation of NCs can alter ligand–ligand (e.g. restraining molecular vibrations), ligand–metal (e.g. varying conformation of interfaces) and metal–metal (e.g. forming metallophilic bonding) interactions, which would further influence the excited state relaxation dynamics. At present, the most widely used methods are the solvent- and cation-induced aggregation of metal NCs triggered by disturbance of solvent polarity and introduction of electrostatic/coordination interactions, respectively. However, both of these approaches are challenging for homogeneity control over the NC aggregates, which in general leads to poor color purity. In contrast, exploring the directed self-assembly of metal NCs is more advanced for aggregating component metal NCs into ordered, customizable and versatile patterns, giving rise to enhanced emission properties with high controllability. Besides, this also provides a platform to unravel the relationship between cluster luminescence and packing attributes (e.g. spatial distribution and inter-NC interactions). In parallel to self-assembly, continuous progress has been made on the crystallization and the scaffold (e.g. zeolites, metal/covalent-organic frameworks) confinement of metal NCs to attain excellent AIE properties [11,12]. Some fundamental inspirations are obtained in these exquisite systems. For example, the metal(I) species-related metal defect state is evidently responsible for the unexpected enhancement of emission intensity of metal NC ensembles. In the crystalline state, metal NCs can show red-shift of the emission band, which is caused by a combined effect of electronic coupling and lattice-originated, nonradiative decay pathways occurring through electron–photon interaction. Moreover, a positive influence of host–guest interaction, polarization effect and the localization of excitons on the emission intensity of metal NCs is observed in a surface confinement system imposed by layered double hydroxide nanosheets.

Although the surface engineering is the most immediate breakthrough in investigation of AIE-type luminescent metal NCs, the contribution of metal(0) core is also important and deserves further attention. The metal(0) core can act as the substrate to anchor surface motifs for their condensation. And, with an oxidation agent, it is able to increase the content of metal(I) species in sacrifice of metal(0) core. In some cases, large metal(0) core (i.e. 2–5 nm) dictated surface plasmon resonance (SPR) can induce a strong coupling effect with the metal(I) involved surface emitter, leading to remarkable emission enhancement of AIE-type metal NCs. Most importantly, the emission states derived from the metal(0) core and the metal(I) surface, generally with a fluorescence and a phosphorescence feature, respectively, can coexist or transform each other in the scheme of AIE-type luminescent metal NCs [1].

To sum up, the AIE mechanism has been widely accepted and continuously improved upon in the research community of luminescent metal NCs. However, the current development of cluster chemistry has not yet reached unambiguous agreement on the AIE fundamentals of metal NC luminescence. In the near future, it is likely that studies on AIE-type metal NCs will remain a research hotspot, which could include but is not limited to:

- Defining the relationship between the structures and the AIE properties of metal NCs to molecular-level precision, which would be greatly beneficial for rational prediction, tailoring and thus customization of highly luminescent metal NCs.
- Tapping the potential of AIE-type metal NCs in applications of related biological systems. The development of water-soluble, small-sized, highly luminescent AIE-type metal NCs, especially with near-infrared (NIR-I and NIR-II regime) absorption and/or emission, is still in its infancy.
- Adding high conductivity, thermostability and processability, etc., to AIE-type metal NCs, thus allowing construction of metal NC-based photoelectric devices.
- Beyond the most investigated noble metals (Au, Ag and their alloys), the earth-abundant, low-cost metals (e.g. Cu, Zn and other transition metals) also deserve extensive exploration. For instance, it would be worth exploring AIE properties in Cu NCs, not only because of their electronic configuration (d^10^s^1^) which is similar to that of Au/Ag, but also because of the wide range of accessible oxidation states (Cu^0^, Cu^I^, Cu^II^, and Cu^III^) [13].
- Understanding of the AIE concept has deepened with increasing structural recognition of luminescent metal NCs. For example, it is more appropriate to use ‘aggregation-induced emission enhancement’ (AIEE) to describe cases that involve the isolated metal NCs with weak emission rather than non-luminescence. Most recently, the AIE of metal NCs has been categorized as clusterization-triggered emission (CTE) in consideration of their totally nonconjugated structures, in which the chromophores in clusteroluminogens are attributed to the significant through-space conjugation (TSC) built in the inter-/intra-NCs [14].

## References

[1] Quintanilla M , Liz-Marzán LM. Science 2018; 361: 645. 10.1126/science.aau5256 30115794

[2] Shang L , Xu J, Nienhaus GU. Nano Today 2019; 28: 100767. 10.1016/j.nantod.2019.100767

[3] Wu Z , Yao Q, Chai OJH et al. Angew Chem Int Ed 2020; 59: 9934–9. 10.1002/anie.202008442 32011796

[4] Mei J , Leung NLC, Kwok RTK et al. Chem Rev 2015; 115: 11718–940. 10.1021/acs.chemrev.5b00263 26492387

[5] Luo Z , Yuan X, Yu Y et al. J Am Chem Soc 2012; 134: 16662–70. 10.1021/ja306199p 22998450

[6] Jadzinsky PD , Calero G, Ackerson CJ et al. Science 2007; 318: 430–3. 10.1126/science.1148624 17947577

[7] Walter M , Akola J, Acevedo OL et al. Proc Natl Acad Sci USA 2008; 105: 9157–62. 10.1073/pnas.0801001105 18599443 PMC2442568

[8] Kang X , Wang S, Song Y et al. Angew Chem Int Ed 2016; 55: 3611–4. 10.1002/anie.201600241 26890334

[9] Zhou M , Higaki T, Hu G et al. Science 2019; 364: 279–82. 10.1126/science.aaw9545 31000661

[10] Pyo K , Thanthirige VD, Kwak K et al. J Am Chem Soc 2015; 137: 8244–50. 10.1021/jacs.5b04210 26061198

[11] Huang R , Wei Y, Dong X et al. Nat Chem 2017; 9: 689–97. 10.1038/nchem.2718 28644463

[12] Chen T , Yang S, Chai J et al. Sci Adv 2017; 3: e1700956. 10.1126/sciadv.1700956 28835926 PMC5562423

[13] Wu Z , Liu J, Gao Y et al. J Am Chem Soc 2015; 137: 12906–13. 10.1021/jacs.5b06550 26397821

[14] Zhang H , Zhao Z, McGonigal PR et al. Mater Today 2020; 32: 275–92. 10.1016/j.mattod.2019.08.010

